# A regulatory focus perspective on eating behavior: how prevention and promotion focus relates to emotional, external, and restrained eating

**DOI:** 10.3389/fpsyg.2014.01314

**Published:** 2014-11-20

**Authors:** Stefan Pfattheicher, Claudia Sassenrath

**Affiliations:** ^1^Institute of Psychology and Education, Ulm UniversityUlm, Germany; ^2^Knowledge Media Research CenterTübingen, Germany

**Keywords:** eating behavior, emotional eating, external eating, prevention focus, promotion focus, regulatory focus, restrained eating

## Abstract

By applying regulatory focus theory (RFT) to the context of eating behavior, the present research examines the relations between individual differences in the two motivational orientations as conceptualized in RFT, that is, prevention-focused and promotion-focused self-regulation and emotional, external, and restrained eating. Building on a representative study conducted in the Netherlands (*N* = 4,230), it is documented that individual differences in prevention focus are positively related to emotional eating whereas negligible associations are found in regards to external and restrained eating. Individual differences in promotion focus are positively related to external eating whereas negligible associations are found in regards to emotional and restrained eating. In relating RFT to different eating styles we were able to document significant relations of basic self-regulatory orientations with regard to essential daily behavior associated with health and well-being. The implications for changing eating styles are discussed.

## INTRODUCTION

Humans differ substantially in terms of eating behavior. For instance, when having experienced negative events some individuals use eating as a strategy to cope with their negative emotions (Macht, 1999; Macht and Simons, 2000). Humans also differ in terms of how much they feel like eating when confronted with food that smells and looks good (Wardle, 1987). Additionally, some individuals have a strong focus on regulating food intake to control body weight (Van Strien et al., 1986). Basically, humans differ regarding both what they eat and how they eat (Young, 1941; Epstein et al., 2007) according to three central dimensions of eating behavior: (a) eating after experiencing negative emotions (i.e., emotional eating, also termed emotional food craving; Hill et al., 1991; Craeynest et al., 2008), (b) eating in response to positive external stimuli such as the smell, taste, and appearance of food (i.e., external eating), and (c) deliberately regulating food intake to control body weight (i.e., restrained eating; Van Strien et al., 1986).

Given that different eating styles are related to important health-related factors such as weight gain and obesity (e.g., Wilson, 1986; Snoek et al., 2007; Baños et al., 2014) it is essential to know *who* engages in what kind of eating styles. Prior research has linked different eating styles to personality dimensions (e.g., Heaven et al., 2001). However, different eating styles have not been analyzed from the perspective of individual differences in self-regulation as conceptualized by a prominent motivational approach: regulatory focus theory (RFT; Higgins, 1997, 1998). In the present contribution, we relate emotional, external, and restrained eating to individual differences in prevention- and promotion-focused self-regulation. In fact, with regard to practical interventions it seems important to know who engages in health-related eating styles, for instance whether emotional eating is likely to be executed by prevention-focused individuals. On this basis one can tailor interventions to fit individuals’ basic self-regulatory orientations. We elaborate on this opportunity in the general discussion.

In the present work, the assumption was put to the test that individuals are more likely to engage in emotional eating the more vigilantly prevention-focused they are. Moreover, it was hypothesized that individuals are more likely to engage in external eating the more promotion-focused they are. No relation was likely to emerge between prevention or promotion orientation and restrained eating. The theoretical notions underlying these assumptions are discussed in the following sections.

### REGULATORY FOCUS THEORY

Human beings (consciously and/or unconsciously) modify and adjust their own habits or actual states to bring these into alignment with a positive standard (e.g., a specific goal in life; Vohs and Schmeichel, 2003; Vohs and Baumeister, 2004). RFT (Higgins, 1997, 1998, 2012a; Scholer and Higgins, 2008, 2011) proposes that it is necessary to differentiate between specific standards (i.e., what is perceived as positive standard) as well as between specific preferred strategies in terms of *how* positively evaluated standards are approached and *how* negatively evaluated standards are avoided. Here, RFT proposes two distinct regulatory systems: a prevention-focused orientation and a promotion-focused orientation (Higgins, 1997, 1998, 2012a; Scholer and Higgins, 2008, 2011). The two basic motivational orientations of prevention focus and promotion focus represent the systems that include the strategies for how individuals approach pleasure and avoid pain. In other words, how individuals generally self-regulate movements toward goals. Prevention-focused individuals typically prefer avoidance strategies in goal striving whereas promotion-focused individuals typically prefer approach strategies (Scholer and Higgins, 2008, 2011; Higgins, 2012b)^1^.

The input factors (i.e., valued standards or reference points) of a prevention focus are safety and security needs. Individuals in a prevention focus are oriented toward significant others, that is, they are concerned with fulfilling duties and responsibilities. Moreover, prevention-focused individuals are motivated to avoid losses and to approach non-losses. In doing so, prevention-focused individuals are sensitive regarding the presence or absence of negative outcomes and information (Scholer and Higgins, 2008, 2011; Higgins, 2012b). Neural correlates support this assumption indicating a greater activity in the amygdala, anterior cingulate, and extrastriate cortex for prevention-focused individuals when negative (vs. positive) information is presented (Cunningham et al., 2005). If a goal is reached, prevention-focused individuals experience quiescence/calmness-related emotions whereas if a goal is missed prevention-focused individuals experience agitation/anxiety-related emotions (Higgins, 1997; Molden et al., 2008).

The input factors of promotion-focused orientation are growth, advancement, and accomplishment. Individuals in a promotion focus are oriented toward ideals, wishes, and aspirations. Promotion-focused individuals are, moreover, motivated to avoid non-gains, and to approach gains. In doing so, promotion-focused individuals are sensitive with regard to the presence or absence of positive outcomes and information (Scholer and Higgins, 2008, 2011; Higgins, 2012b). Neural correlates also support this assumption indicating greater activity in the amygdala, anterior cingulate, and extrastriate cortex for promotion-focused individuals when positive (vs. negative) information is presented (Cunningham et al., 2005). If a goal is reached, promotion-focused individuals experience cheerfulness/happiness-related emotions whereas if a goal is missed promotion-focused individuals experience dejection/sadness-related emotions (Higgins, 1997; Molden et al., 2008).

Regulatory focus theory postulates that promotion focus represents a distinct orientation and is not the opposite orientation to prevention focus (Higgins, 1997, 2012a). This suggests that it is possible that one of the two orientations is associated with a certain external construct whereas the other orientation is not. This is relevant in the present context given that specific eating styles are expected to be related to one motivational orientation while the other motivational orientation may not be related.

### REGULATORY FOCUS AND EATING BEHAVIOR

In the context of eating behavior, Florack et al. (2013) have shown that prevention-focused (vs. promotion-focused) individuals ensure appropriate eating behavior by following the eating behavior of others^2^. In another study it was found that prevention-focused individuals consumed more fruits associated with health precautions than associated with benefits (Spiegel et al., 2004). Joireman et al. (2012) documented that the more promotion-focused an individual was the more likely they were to report eating healthily in order to feel good. Recently, Pula et al. (2014) examined the relations of regulatory focus and food choice motives, showing that the prevention focus is associated with emphasizing mood, convenience, and familiarity. However, the relations between specific eating styles and chronic prevention and promotion focus have not been examined before. Therefore, we now outline in detail how different eating styles, in particular emotional, restrained, and external eating, are expected to be related to prevention and promotion focus.

Eating after experiencing negative emotions (i.e., emotional eating) is considered to be a response to cope with negative events and the resulting negative emotions when they cannot be appropriately regulated in a more adaptive way (Arnow et al., 1995; Macht and Simons, 2000; Evers et al., 2010). That is to say, emotional eating represents a coping style that reflects individuals’ motivation to avoid a negative emotional state; so emotional eating is associated with the avoidance system (Cochrane et al., 1992; Spoor et al., 2007). Emotional eating is closely related to food cravings both conceptually and empirically (Hill et al., 1991; Craeynest et al., 2008; Rodríguez-Martín and Molerio-Pérez, 2014), especially the food cravings that occur after an individual experiences negative events and emotions (Nijs et al., 2007; Meule et al., 2012). Correspondingly, strong emotional eaters eat more snack foods compared with weak emotional eaters (de Lauzon et al., 2004), especially when in a state of distress or sadness (Van Strien et al., 2012, 2013).

Regarding the relation between emotional eating and regulatory focus, we build on the notion that emotional eating can be conceptualized as an avoidance strategy to cope with negative events and emotions (Cochrane et al., 1992; Spoor et al., 2007). Specifically, emotional eating reflects a tendency to avoid a negative emotional state in order to approach a more positive emotional state. This is particularly relevant in relation to prevention focus given that prevention-focused individuals are typically sensitive to negative events and typically use avoidance strategies to cope and to approach a more general positive state (Scholer and Higgins, 2008, 2011; Higgins, 2012b; Keller and Pfattheicher, 2013; Pfattheicher and Keller, 2013; Cheung et al., 2014). In line with these considerations, Pula et al. (2014) showed that prevention-focused individuals emphasize the mood regulating function of food regarding negative states. On this basis we assume that prevention-focused individuals are most likely to engage in emotional eating, that is, a positive association between individual differences in prevention focus and emotional eating is expected.

External eating represents eating in response to positive external stimuli such as the smell, taste, and appearance of food. In short, external eating does not reflect a coping strategy for negative emotions (as emotional eating does) but rather an approach to attractive food when it is present that may result in over-eating (Van Strien et al., 2009). Building on the notion that external eating can be conceptualized as an approach strategy to attain positive external stimuli (i.e., favorable food), and promotion-focused individuals are typically sensitive to positive stimuli, and use approach strategies to ensure their wishes (Scholer and Higgins, 2008, 2011; Higgins, 2012b), we assume that promotion-focused individuals are more likely than others to engage in external eating. That is, a positive association between individual differences in promotion focus and external eating is expected.

Restrained eating implies that individuals regulate their food intake with regard to weight control. As such, restrained eating is more likely to be displayed by individuals with a relatively high BMI (Heaven et al., 2001; Provencher et al., 2003; Elfhag and Linné, 2005; Snoek et al., 2007, 2013; but see Baños et al., 2014). Also, restrained eaters eat more unhealthy food such as sweets (Elfhag et al., 2008).

Regarding the relation between restrained eating and regulatory focus, Vartanian et al. (2006) report no significant relations between restrained eating and individual differences in prevention and promotion focus. Indeed, it is not likely that the *general* notion of restrained eating reflects a *specific* orientation of regulatory focus. Restrained eating can be framed as approaching a positive outcome (losing weight), that is, it can fit a promotion-focused strategy. However, restrained eating can also be framed as avoiding a negative outcome (gaining weight), that is, it can fit a prevention-focused strategy. In the study reported below, however, only general restrained eating is assessed (e.g., “How often do you try not to eat over the course of an evening because you are dieting?”) which does not include whether individuals focus on a positive outcome (approaching losing weight) or a negative outcome (avoiding gaining weight) when engaging in restrained eating. Accordingly, it seems unlikely for *general* restrained eating to be related to *specific* motivational orientations (i.e., prevention or promotion focus). These considerations are in line with the study by Vartanian et al. (2006) which documents null relations.

In sum, the current study investigated whether individuals are more likely to engage in emotional eating the more prevention-focused they are. Moreover, it was tested whether individuals are more likely to engage in external eating the more promotion-focused they are. In line with the findings of Vartanian et al. (2006), no relations were expected to emerge between prevention or promotion focus and restrained eating.

## STUDY

### METHOD

#### Participants

The study involves a representative study (the LISS panel) conducted in the Netherlands (*N* = 4,230; *M*_age_ = 52.29; 53.4% women). In this study, we took advantage of the panel-character of the LISS panel which allows the merging of several waves of the panel. In February 2011, individual differences in prevention and promotion focus were assessed; in July 2010, The Dutch Eating Behavior Questionnaire (Van Strien et al., 1986) was utilized to measure emotional, external, and restrained eating. Alpha reliabilities, means, and standard deviations are displayed in **Table 1**.

**Table 1:** Alpha reliabilities, means, standard deviations (on the diagonal) and zero-order correlations.

	Prevention	Promotion	Emotional eating	Restrained eating	External eating	BMI
Prevention	1.00					
Promotion	0.54****	1.00				
Emotional eating	0.24****	0.16****	1.00			
Restrained eating	0.10****	0.09****	0.28****	1.00		
External eating	0.16****	0.23****	0.56****	0.11****	1.00	
BMI	0.00	-0.07****	0.13****	0.19****	0.01	1.00
Mean	3.25	3.86	2.02	2.74	2.67	25.63
SD	1.13	1.20	0.74	0.79	0.46	4.63
α	0.85	0.90	0.92	0.96	0.83	-

****p < 0.0001; Prevention and Promotion were assessed on 7-point Likert scale, eating styles on a 5-point Likert scale.

#### Regulatory focus

Chronic self-regulatory orientations were assessed using a Dutch version of the regulatory focus scale (RFS) developed by Lockwood et al. (2002). A sample item of the 9-item prevention focus subscale reads: “In general, I am focused on preventing negative events in my life.” A sample item of the 9-item promotion focus subscale reads: “I frequently imagine how I will achieve my hopes and aspirations.” The scale endpoints of the items were labeled “1” (not at all true) and “7” (completely true).

#### Eating behavior

The Dutch Eating Behavior Questionnaire (Van Strien et al., 1986) assessed emotional, restrained, and external eating. A sample item of the 13-item emotional eating subscale reads: “Do you have a desire to eat when you are emotionally upset?” A sample item of the 10-item restrained eating subscale reads: “Do you deliberately eat things that are good in terms of weight control?”^3^ A sample item of the 10-item external eating subscale reads: “If you walk past the bakery do you have the desire to buy something delicious?” The scale endpoints of the items were labeled “1” (never) and “5” (very often).

#### BMI

The LISS panel also includes a self-report of height and weight on the basis of which participants’ BMI was calculated. BMI was included because of its associations with different eating styles (e.g., Baños et al., 2014) and to show relations of regulatory focus and eating styles beyond BMI.

## RESULTS

Zero-order correlations among the applied constructs are displayed in **Table 1**. These revealed significant positive associations (*p* < 0.0001) between emotional eating and restrained eating as well as external eating. External eating and restrained eating were negligibly correlated. The strength of these correlations was comparable to other research (e.g., Ellickson-Larew et al., 2013). In the present sample, BMI had the strongest positive correlation with restrained eating (*r* = 0.19; see also **Table 1**). BMI was less strongly correlated with emotional eating (*r* = 0.13) and was not significantly correlated with external eating (*r* = 0.01).

Regarding prevention focus, zero-order correlations revealed that prevention focus had the strongest correlation with emotional eating. Promotion focus had the strongest correlation with external eating. In this sample, individual differences in promotion and prevention were also correlated (*r* = 0.54, *p* < 0.0001)^4^. Thus, zero-order correlations with one self-regulatory orientation may be biased due to the shared variance with the respective other self-regulatory orientation. Therefore, multivariate analyses were applied. These analyses revealed that individuals are more likely to engage in emotional eating the more prevention-focused they are in their orientation (β = 0.22, *p* < 0.0001; see **Table 2**, Model 1). The relation between emotional eating and promotion focus was negligible (β = 0.04), although still significant (*p* < 0.05). For external eating, individuals are more likely to engage in this eating behavior the more promotion-focused they are in their orientation (β = 0.20, *p* < 0.0001). The relation between external eating and prevention focus was negligible (β = 0.06), although still significant (*p* < 0.01). No substantial (but still significant) relations between prevention focus or promotion focus and restrained eating were found (βs < 0.08). These relations also hold when including the BMI factor in the analyses (**Table 2**, Model 2) and when controlling for the respective two other eating behaviors (e.g., controlling for restrained and external eating when predicting emotional eating; see **Table 2**, Model 3).

**Table 2:** Multivariate OLS regression analyses.

	Emotional eating						External eating						Restrained eating					
	Model 1		Model 2		Model 3		Model 1		Model 2		Model 3		Model 1		Model 2		Model 3	
B	β	B	β	B	β	B	β	B	β	B	β	B	β	B	β	B	β
Constant	1.46****		0.90****		-1.29****			2.30****		2.24****		1.97****			2.45****		1.60****		1.57****	
Prevention	0.14****	0.22	0.14****	0.21	0.11****	0.166		0.02**	0.06	0.02**	0.06	-0.02****	-0.06		0.06****	0.08	0.05****	0.07	0.01*	0.01
Promotion	0.03*	0.04	0.03**	0.05	-0.04****	-0.063		0.08****	0.20	0.08****	0.20	0.07****	0.17		0.03*	0.04	0.04***	0.06	0.38**	0.06
BMI			0.02****	0.133	0.01****	0.083				0.00	0.02	-0.00****	-0.04				0.03****	0.19	0.03****	0.15
Emotional eating												0.35****	0.57						0.30****	0.29
External eating					0.85****	0.525													-0.11****	-0.06
Restrained eating					0.19****	0.197						-0.03****	-0.05							
F	129.84****	114.90****	540.99****		120.47****	81.14****	437.62****		25.16****	70.57****	103.417****
R	0.06	0.08	0.39		0.05	0.05	0.34		0.01	0.05	0.11
		


*p < 0.05, **p < 0.01, ***p < 0.001, ****p < 0.0001; all SEs were below 0.03.

## DISCUSSION

In relating RFT to different eating styles we were able to document significant relations of basic self-regulatory orientations with essential daily behavior associated with health and well-being (Van Strien et al., 1986; Wilson, 1986; Snoek et al., 2007; Alberts et al., 2012; Baños et al., 2014). Specifically, the present work examined the relation between different eating styles, in particular emotional, external eating, and restrained eating and prevention-focused and promotion-focused self-regulation. Analyses revealed that individual differences in prevention focus were positively related to emotional eating. In this regard, a medium effect size was found (Cohen, 1988). That is to say, the more individuals are chronically prevention-focused the more they use emotional eating to cope with negative emotions and events. Moreover, individual differences in promotion focus were positively related to external eating. Here, a medium effect size was found, too (Cohen, 1988). This strengthened the assumption that external eating reflects an approach type of behavior – behavior that is executed in particular by promotion-focused individuals. Regarding restrained eating, negligible associations with prevention and promotion focus are found. In sum, the present research contributes to the field of self-regulation, specifically to research on regulatory focus and extends existing knowledge about how basic motivation orientations as conceptualized in RFT relate to eating behavior (Spiegel et al., 2004; Joireman et al., 2012; Florack et al., 2013).

In critically reflecting upon the present work, we point to the fact that the reported data is of a correlational nature. Consequently, causation should not be assumed. Meanwhile, different directions of the observed correlations could be possible, for instance that prevention focus is the result and not the cause of emotional eating. That is to say, after engaging in emotional eating individuals may use vigilant, prevention-focused strategies to deal with the negative event of emotional eating which may have included over-eating and food cravings for unhealthy food.

Further research should address the causal nature of the associations by, for instance, manipulating the respective self-regulatory system (Shah et al., 1998; Friedman and Förster, 2001; Freitas et al., 2002).

Going back to the theoretical approach of RFT, the two modes of self-regulation (prevention and promotion) have been conceptualized as distinct orientations (Higgins, 1997, 2012a). Thus, prevention-focused self-regulation does not represent the opposite of promotion-focused self-regulation. This suggests that one of the two orientations can be associated with a certain construct whereas the other orientation is not, which is precisely what was found. Prevention focus predicted emotional eating whereas no substantial relation was found with promotion focus. Congruently, promotion focus predicted external eating while prevention focus was not substantially related to this eating style. That is to say, the present findings are in line with the distinctness assumption of RFT as documented in other research (e.g., Scholer et al., 2010; Keller and Pfattheicher, 2011).

We want to emphasize that the relation between prevention focus and emotional eating actually is not as straight-forward as it may seem. Prevention focus is occasionally conceptualized as a defensive mode of self-regulation sharing substantial communalities with behavioral avoidance and inhibition (e.g., Förster et al., 1998, 2001). This is especially the case if conceptualizing regulatory focus more concrete as self-regulation following the attainment of different standard (i.e., approaching a positive state, a ‘gain,’ in a promotion focus and avoiding a state, a ‘loss,’ in the prevention focus) as it relates promotion focus and approach and prevention focus and avoidance to some extent (see Summerville and Roese, 2008, for an overview). Yet, the present study suggests that prevention-focused individuals actively go for food in order to cope with negative emotions. Additionally, if prevention-focused individuals typically engage in behavioral avoidance and inhibition prevention focus should be positively related to the defensive eating style of restrained eating. This is, however, not the case. Prevention focus is very weakly related to restrained eating (see **Table 2**). These findings are incongruent to the notion that prevention focus mainly reflects behavioral avoidance and inhibition. As such, the present work also contributes to research on regulatory focus.

The present work is also relevant in terms of implications for changing eating styles. One can state that prevention-focused individuals are sensitive regarding the presence or absence of negative states and information (Scholer and Higgins, 2008, 2011; Higgins, 2012b). In order to reduce chronically prevention-focused individuals’ emotional eating one could stress the negative consequences of emotional eating. In fact, it is documented that emotional eating results in weight-gain (Van Strien et al., 2012, 2013). In this way one may use prevention-focused individuals’ sensitivity to negative information to reduce their use of emotional eating to cope with negative events. Regarding promotion focus, one can state that promotion-focused individuals are sensitive regarding the presence or absence of positive states and information (Scholer and Higgins, 2008, 2011; Higgins, 2012b). On this basis one could emphasize that external eating can lead to reduced healthiness (i.e., the absence a positive state). For instance, it is shown that external eating is related to overeating (Van Strien et al., 2009). In this way one may use promotion-focused individuals’ sensitivity to the absence and presence of positive information to reduce their use of external eating.

Another possibility to change eating styles is offered by Evers et al. (2010) and Alberts et al. (2012). Alberts et al. (2012) reduced vigilant avoidance strategies through the use of a mindfulness intervention. Mindfulness fosters the acceptance of a current (negative) state thus reducing vigilance and avoidant goal striving. Results show that being mindful reduces food cravings when experiencing negative emotions, that is, mindfulness leads to less emotional eating (see also Alberts et al., 2010). Other work by Evers et al. (2010) shows that reappraisal rather than maladaptive suppression of negative emotions leads to less emotional eating. In sum, these studies show that reducing the vigilant avoidance system leads to less emotional eating. Building on our research, one could expect that these interventions would be particularly effective for individuals who actually use vigilant avoidance strategies (i.e., prevention-focused individuals). As such, mindfulness training and interventions to reappraise negative emotions can be especially useful for chronically prevention-focused individuals to regulate their food cravings.

Congruently, one might also aim to change external eating behavior, an important endeavor as external eating can result in over-eating (Van Strien et al., 2009) or maladaptive night eating (Nolan and Geliebter, 2012). In this regard, one can implement strategies for promotion-focused individuals to reduce external eating. Specifically, when aiming to reduce external eating one may focus on the reduction of promotion-focused individuals’ approach tendencies when attractive food is present or can be reached. To this end, one could diminish the attractiveness of food (Visschers and Siegrist, 2009) so that chronically promotion-focused individuals’ are less attracted by the food. In sum, we propose that interventions should be designed to fit individuals’ basic self-regulatory orientations.

To conclude, the present work reflects a fruitful approach for future research examining the impact of self-regulatory orientations on emotional, restrained, and external eating. As such, the present work provides new impulses for the design of effective interventions aiming at reducing maladaptive eating styles.

## Conflict of Interest Statement

The authors declare that the research was conducted in the absence of any commercial or financial relationships that could be construed as a potential conflict of interest.

## Footnotes

1 As this work is situated in the context of food cravings and restrained eating it is important to distinguish between self-regulation as conceptualized by RFT and self-regulation in terms of self-control resources. Self-regulation in terms of self-control resources means that individuals forgo short-term gratification in service of higher ordered long-term goals. Closely related to this is research on ego-depletion showing when individuals’ self-control resources are depleted they seek short-term gratification (e.g., Baumeister et al., 1998). That is to say, individuals regulate themselves using self-control resources. Our work, however, does not build on self-regulation in terms of self-control, ego-depletion, and appealing short-term gratification. The notion of self-control resources and ego-depletion is not explicitly implemented in RFT (see Higgins, 2012a). Rather, the present work focuses on the general strategies individuals use to approach pleasure and avoid pain, that is, how individuals generally regulate movements toward goals (Higgins, 1997). In contrast to the self-control resource approach (Baumeister et al., 1998), RFT does not emphasize a trade-off between short-term and long-term goals. One can use prevention and promotion strategies to move toward short-term and long-term goals. Additionally, RFT emphasizes that it is necessary to differentiate between specific standards, that is, what is perceived as a pleasure or positive standard (e.g., prevention-focused individuals emphasize safety and security needs). What is perceived as a pleasure or positive standard is not implemented in the self-control resource approach (Baumeister et al., 1998). Neither are basic needs and relevant standards implemented in this approach, in contrast to RFT. In sum, the two meanings of self-regulation represent distinct conceptualizations.

2 We note that prevention focus is not the opposite of promotion focus. Therefore, one cannot conclude whether prevention or promotion focus drives the effects.

3 We note that one item of the scale is framed in a prevention-oriented way (“Do you deliberately eat less in order not to become heavier?”). Results remain exactly the same when excluding this item.

4 We want to point to the relatively strong positive correlation between prevention and promotion focus which is higher compared with other samples involving students (cf. Keller and Pfattheicher, 2013). This finding could be due to the specificity of this sample (representative sample with older adults instead of a student population) and speaks to the notion that prevention and promotion focus strategies are more strongly combined later in life and that older individuals are more likely to use prevention and promotion strategies in a more balanced way (resulting in a stronger positive correlation) whereas in younger individuals one strategy seems to predominate (resulting in a relatively weak positive correlation). In fact, using the younger respondents, that is, the age group of 16–25 results in a significantly (*p* < 0.001) weaker correlation (*r* = 0.19, *p* < 0.001; *n* = 373) compared to the entire sample (*r* = 0.54; *n* = 4,230). Additionally, we found a linear positive association between age and the strength of the prevention-promotion correlation (from 0.47, *p* < 0.001 in the age group of 35–45; *n* = 563 up to 0.63, *p*< 0.001 in the age group of 65–75; *n* = 454). Accordingly, the strong correlation seems to reflect an age effect. These findings are in line with previous research on regulatory focus and aging (Lockwood et al., 2005).
